# Evaluation of Storage Stability of Date Fruit (*Phoenix dactylifera* L.) at Two Levels of Relative Humidity Based on Selected Functional Compounds and Image Features

**DOI:** 10.3390/foods14183189

**Published:** 2025-09-12

**Authors:** Younes Noutfia, Ewa Ropelewska, Justyna Szwejda-Grzybowska, Zbigniew Jóźwiak, Sebastian Siarkowski, Monika Mieszczakowska-Frąc, Krzysztof P. Rutkowski

**Affiliations:** Fruit and Vegetable Storage and Processing Department, The National Institute of Horticultural Research, Konstytucji 3 Maja 1/3, 96-100 Skierniewice, Poland; justyna.grzybowska@inhort.pl (J.S.-G.); zbigniew.jozwiak@inhort.pl (Z.J.); sebastian.siarkowski@inhort.pl (S.S.); monika.mieszczakowska@inhort.pl (M.M.-F.); krzysztof.rutkowski@inhort.pl (K.P.R.)

**Keywords:** ‘Mejhoul’, relative humidity, polyphenols, sugars, color, prediction

## Abstract

In this study, two known date fruit cultivars (‘Mejhoul’ and ‘Boufeggous’) were pre-treated using convective and infrared drying, and then subjected to cold storage at +4 °C using two levels of relative humidity (RH): 55% and 65%. The quality of the date fruit was assessed based on selected phenolic compounds, sugars, color, hardness, weight loss, and some image features at 0, 2, 4, 6, and 8 months. The results exhibited non-similar patterns in the changes of phenolic compounds according to cultivar and relative humidity level. The highly significant changes were observed for “Quercetin-Xyloside” that decreased drastically after two months of storage, especially for the ‘Mejhoul’ cultivar. Also, “Gallic acid” indicated a progressive and significant increase during storage. Other phenolic compounds, mainly “Quercetin-derivatives”, “Chlorogenic acid derivatives”, and “Ferulic acid”, showed fluctuating values among treatments and during the whole period of storage. For sugar compounds, it was noticed that glucose and fructose were affected by drying technique and relative humidity, especially for the ‘Boufeggous’ cultivar. Weight loss increased significantly for ‘Mejhoul’ cultivar stored at high relative humidity (65%), and ‘Boufeggous’ evolved in the opposite way for samples stored at lower RH (55%). For color, the browning index exhibited a significant and progressive increase among all treatments considered in this study; this pattern was more pronounced for date cultivars stored at high relative humidity (65%). The evaluation of hardness indicated a softening phenomenon during storage, especially for the ‘Mejhoul’ cultivar stored at 65% of RH. Moreover, the correlation of image features with weight loss and some color attributes indicated high correlation, and the estimation of the behavior of dates under storage could be predicted using accurate image features.

## 1. Introduction

The perishability of fruit represents a serious challenge to farmers, the fresh product supply chain, as well as consumers [1]. Date fruit (*Phoenix dactylifera* L.) is among the highly perishable stone fruits, especially for soft cultivars or for cultivars harvested at earlier or at middle stage of maturity [2,3]. As a climacteric fruit, date fruit ripens rapidly after harvesting, leading in several cases to subsequent market losses [4]. Thus, several approaches based on farm monitoring, mastery of agricultural practices (irrigation, fertilization, etc.), as well as application of appropriate postharvest operations such as polishing, hydration, and drying, are generally applied to lease a high-quality fresh date fruit in the markets [5]. However, these approaches remain insufficient when the need to ensure the availability of date fruit in the market for a long time. Therefore, numerous preservation techniques were suggested to maintain the freshness and quality of date fruit as well as the storage stability after harvesting [6]. The main preservation techniques are chilling, freezing, modified and controlled atmosphere packaging, edible coating, ultrasonication, and application of chemical reagents [7,8,9,10].

Cold storage techniques are considered among the highly effective methods to extend the shelf-life of several date cultivars at different maturity stages [6]. Those techniques stop or delay the ripening process, the physiological reactions such as respiration, senescence, and preserve the total quality. Two parameters are critically important during cold storage: temperature and relative humidity. The optimal range of temperature is between 0 and 4 °C, depending on cultivar, the softening level, and the desired period of storage. Regarding relative humidity (RH), appropriate levels of 65–75% were suggested with some flexibility regarding the water activity of date samples [11].

RH is defined as the moisture content of the atmosphere expressed as a percentage of the amount of moisture that can be retained by the atmosphere (at a defined temperature and pressure) without condensation. RH is a critical factor that impacts the water loss during storage, the microbial growth, and the physicochemical quality attributes [11,12].

In the available literature, several works have focused on assessing the behavior of date samples by controlling only the temperature factor. Thus, it was found that the storage of two Moroccan date cultivars at 2–4 °C (without controlling relative humidity) leads to significant changes in the main physicochemical attributes with different behavior according to the cultivar [13]. In another investigation, Bisir ‘Barhi’ date fruit was stored at 1 °C and at 90–95 RH, showing increases in weight loss with fluctuation in phenolic compounds and antioxidant activity [4]. Moreover, a recent study suggested the temperature of +2 °C as the optimal temperature for the storage of ‘Deglet Nour’ cultivar without any recommendation regarding RH [14]. Similarly, it was highlighted that the preservation of soft and semi-soft date fruit at the range of +4 °C to +10 °C could prevent some undesirable physicochemical reactions during storage and maintain good date fruit quality [15]. The previously mentioned studies did not suggest any range for relative humidity, considered as a powerful parameter in the preservation of date fruit quality, especially soft cultivars [11]. In addition, the available literature for date (*Phoenix dactylifera* L.) fruit was interested in evaluating the physicochemical characteristics and, in some cases, the functional compounds of date fruit during storage based on total polyphenols and flavonoids using classical and destructive methods. Thus, only a few studies have controlled relative humidity during the cold storage of date fruit and are interested in profiling the individual phenolic compounds.

In this perspective, the relevance of our investigation (in comparison with available literature) is to evaluate the effect of long-term cold storage at +4 °C using two levels of relative humidity: 55% and 65%. In addition, this investigation aims to carry out an in-depth analysis of polyphenolic compounds, sugars, and some physical attributes during storage. Moreover, this work will establish a correlation between the mentioned quality attributes and image features obtained in a non-destructive manner in order to predict the behavior of date fruit under storage. Likewise, the use of image features could bring comprehensive insights into the storage behavior of date fruit by linking those features to key physicochemical characteristics of date fruit that impact both nutritional and commercial quality.

## 2. Materials and Methods

### 2.1. Plant Material

100 kg of full-mature ‘Mejhoul’ and ‘Boufeggous’ date cultivars were collected randomly from a Moroccan orchard located in the Southern East of Morocco. Both date fruit cultivars were packed in carton boxes, transported by air to the Fruit and Vegetable Storage and Processing Department of the National Institute of Horticultural Research (INHORT, Skierniewice, Poland), and stored at 2–4 °C for 24 h before sorting and carrying out drying and storage experiments. Manual sorting was carried out in order to select only date fruit with homogeneous color, without visual defects, and with similar shape.

### 2.2. Prior Drying Experiments

To improve the external quality of date fruit, reduce the possibility of mechanical damage, and enhance the cold storage operation by slightly reducing the initial moisture content, prior drying was applied to both date cultivars using convective and infrared drying. Thus, date samples (with stone) were spread in trays in one layer and subjected to drying at 60 °C in the CONVECO industrial dryer (CONVECO Sp. z o. o., Glinianka, Poland). The drying treatment was applied using two technological repetitions, and the analytical conditions were as described by Noutfia et al. [16]. After drying, date fruit samples were kept at room temperature for 12 h before starting cold storage experiments.

### 2.3. Cold Storage

Both dried cultivars were transferred to small PET boxes and stored at +4 °C/55% of relative humidity (RH) and at +4 °C/65% of RH during 8 months of storage. The choice of this range [55–65%] is justified by the range of water activity found for each date cultivar that was between 0.561 and 0.647 (Table 1). When the water activity of the raw material subjected to storage is close to the relative humidity of the atmosphere, the phenomenon of weight loss is reduced and exchanges are limited.

**Figure 1:**
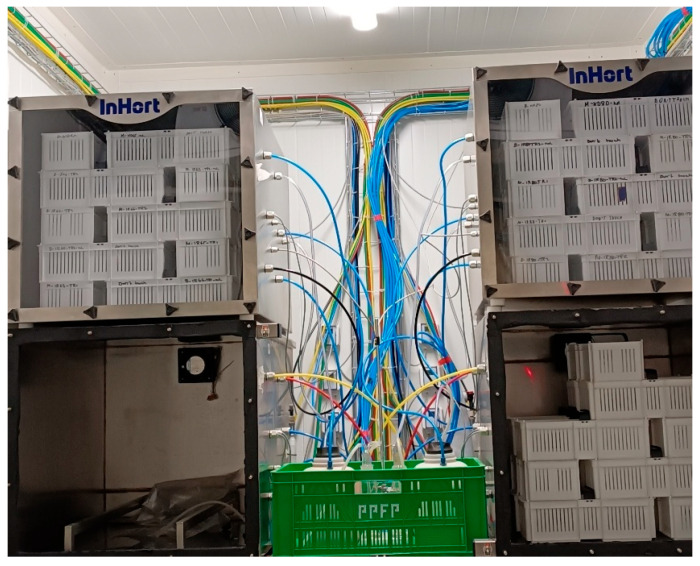
Illustration of the storage conditions of date fruit at the two selected relative humidity levels.

The experimental design for storing date fruit at the two selected relative humidity levels is given in Figure 1. The quality of stored date fruit samples was assessed at 0, 2, 4, 6, and 8 months of storage. For polyphenols and sugars, the assessment was realized at 0, 4, and 8 months.

In this investigation, eight treatments are considered:

M-CD55: Convective dried ‘Mejhoul’ stored at +4 °C/55% of RH.

M-CD65: Convective dried ‘Mejhoul’ stored at +4 °C/65% of RH.

M-IRD55: Infrared dried ‘Mejhoul’ stored at +4 °C/55% of RH.

M-IRD65: Infrared dried ‘Mejhoul’ stored at +4 °C/65% of RH.

B-CD55: Convective dried ‘Boufeggous’ stored at +4 °C/55% of RH.

B-CD65: Convective dried ‘Boufeggous’ stored at +4 °C/65% of RH.

B-IRD55: Infrared dried ‘Boufeggous’ stored at +4 °C/55% of RH.

B-IRD65: Infrared dried ‘Boufeggous’ stored at +4 °C/65% of RH.

### 2.4. Sugar Analysis

Sugars were analyzed by high-performance liquid chromatography on an Agilent 1200 HPLC system, equipped with a differential refractometric detector, using Aminex HPX-87C (300 mm × 7.5 mm) with a mobile phase of 0.1 N edetate calcium disodium (CaNa2-EDTA) at an isocratic flow of 0.6 mL min^−1^, and a temperature of 80 °C. Results were expressed in g 100 g^−1^ of dry mass (DM).

### 2.5. Phenolic Compounds

In both date cultivars, phenolic compounds were determined according to a modified high-performance liquid chromatography method using an Agilent 1200 HPLC system equipped with a diode array detector [17]. The calculations of phenolic compounds were quantified according to calibration curves determined with standards for individual polyphenols from the groups of flavan-3-ols, hydroxybenzoic acids, hydroxycinnamic acids, flavonols, and flavones, and results were expressed as mg 100 g^−1^ of DM.

### 2.6. Color, Hardness, and Weight Loss

The color was analyzed at each storage time for 20 date fruit samples using a spectrophotometer (Konica Minolta CM-2600d, Chiyoda, Tokyo, Japan). The color parameters L* (lightness), a* (green-red), and b* (blue-yellow) were automatically recorded. For each fruit, two readings per date fruit were taken on opposite sides.

For hardness, 25–30 pitted date samples were subjected to punch assay using an Instron 4303 texturometer (Instron Corp., Norwood, MA, USA), and the maximal load, expressed in Newton, was recorded.

Regarding weight loss, it was analysed on the same fruit samples prior to and at each storage time. For each treatment, 100 fruit samples were employed, and the determination of weight loss/gain was achieved as follows: Weight loss rate (%) = [(W0 − W1)/W0] × 100 %, where (W0) corresponds to the initial fruit weight and (W1) is the weight after the specified storage period.

### 2.7. Image Analysis

Images of date fruit (resulting from all considered treatments) were acquired using a Canon digital camera (Canon EOS 2000D, Ōta, Tokyo, Japan) with a Canon Zoom Lens EF-S 18–55 mm/Ø58 mm. After image acquisition, the background was changed to black. Additionally, image segmentation was carried out using Q-MAZDA 23.10 software in the objective to determine the regions of interest (Łódź University of Technology, Institute of Electronics, Łódź, Poland) [17,18]. Then, image features were extracted based on color, texture, and morphological features, allowing the obtention of 599 image features for each ROI.

### 2.8. Statistical Analysis and Correlation Analysis

The mean comparison and correlation analysis were performed using STATISTICA 13.3 (StatSoft Polska Sp. z o.o., Kraków, Poland, TIBCO Software Inc., Palo Alto, CA, USA). A one-way analysis of variance (ANOVA) was carried out to compare the mean values of physicochemical properties and determine the statistically significant differences between the analyzed groups of date fruit. In this order, the variance, homogeneity, and normality of the distribution were checked. Finally, Tukey’s test at *p* < 0.05 was used. Pearson’s correlation coefficients (R) at *p* < 0.05 and related graphs were determined to reveal the linear relationships between image textures and selected physicochemical parameters of date fruit.

## 3. Results

### 3.1. Physicochemical Properties of Dried Date Fruit Before Storage

The main physicochemical attributes of convective and infrared dried ‘Mejhoul’ and ‘Boufeggous’ date samples are reported in Table 1.

The performed analysis demonstrated the effect of drying technique on the majority of physicochemical properties of both cultivars. The total polyphenols, total flavonoids, and total phenolic acids contents were significantly higher in convective dried samples for both date cultivars. In the case of TP, the obtained values were respectively 37.1 and 31.7 for convective and infrared dried ‘Mejhoul’; compared to only 29.9 and 24.9 for convective and infrared dried ‘Boufeggous’. For sugars, no significant differences were observed between convective and infrared dried date samples, besides the Glucose content of the ‘Boufeggous’ cultivar, which showed lower content for infrared dried samples. For TSS, no significant effect of drying technique was recorded for the case of ‘Boufeggous’ compared to ‘Mejhoul’, which exhibited significantly higher values for infrared-dried date samples. Water activity remained without any significant changes between the two applied drying techniques for both cultivars. For hardness, the values of 9.26 N and 9.13 N were recorded respectively for convective and infrared dried ‘Mejhoul’ compared to only 5.45 and 4.50 for ‘Boufeggous’ subjected to the respective drying techniques. For color parameters, the yellowness (b*) seems not to be affected by drying compared to L* and a*, which exhibited significant differences according to date cultivar and drying technique.

### 3.2. Sugar Profile Under Storage

The assessment of sugar profile based on glucose, fructose, and total sugars during cold storage is given in Table 2.

In the present study, the sugar content of ‘Mejhoul’ and ‘Boufeggous’ at three times (0, after 4, and 8 months) of cold storage was measured based on total sugars, glucose, and fructose. As shown in Table 2, the variation of sugars during storage depended mainly on the cultivar and the relative humidity of the storage atmosphere.

For the ‘Mejhoul’ cultivar stored at 55% of relative humidity, it was found that the content of total sugars, glucose, and fructose decreased significantly after 4 months of cold storage before increasing at the end of storage. As an illustration of this trend, the total sugar concentration of “M-IRD55” was 83.5 g 100 g^−1^ DM at the beginning of storage, reaching a minimum value of 80.4 g 100 g^−1^ DM at M4, before increasing to 83.8 g 100 g^−1^ DM at M8. For the same cultivar (and at a higher relative humidity of 65%), it was found that both convective and infrared dried date samples of the ‘Mejhoul’ cultivar showed a higher concentration of all analyzed sugars at the beginning of storage before a significant decrease at M4. Also, the data exhibited no significant differences between M4 and M8. For the example of “M-CD65”, the initial content of glucose was 41.6 g 100 g^−1^ DM, before a significant decrease to 40.5 at M4 and to 40.4 g 100 g^−1^ DM at M8.

For ‘Boufeggous’, the changes in sugar profile were affected during storage by drying technique and relative humidity. Also, those changes were more pronounced and significant for glucose compared to fructose and total sugars. In this context, a continuous significant increase in glucose content during storage was reported for “B-CD55”, “B-CD65”, and “B-IRD55”; however, no significant changes were found for the treatment “B-IRD65”. For fructose, no significant changes were detected in this sugar component for ‘Boufeggous’ stored at 65% compared to date samples stored at 55%. At these lower relative humidity conditions, a significant increase in fructose content was observed since M4 for “B-IRD55” and later at M8 for “B-CD55”. Moreover, the results reported in Table 2 exhibited a similar trend in the evolution of total sugars as those detailed for fructose rather than glucose. The biggest changes in total sugars among stored ‘Boufeggous’ treatments were shown for the case of infrared-dried ‘Boufeggous’ stored at 55% of relative humidity. In this case, the amount of total sugars exhibited a continuous significant increase from 78.8 to 82.2 and 85.6 g 100 g^−1^ DM for M0, M4, and M8, respectively.

### 3.3. Polyphenolic Compounds Under Storage

**Figure 2:**
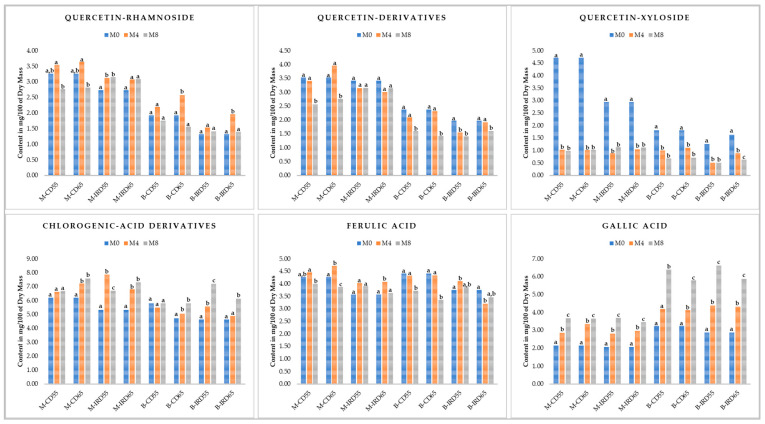
Evolution of main phenolic compounds of convective and infrared dried ‘Mejhoul’ and ‘Boufeggous’ subjected to two different storage conditions of 55% and 65% of relative humidity. For each phenolic compound and each block of experiments, different letters refer to significant difference (among months of storage) by Tukey test (*p* < 0.05).

The major phenolic compounds found previously in ‘Mejhoul’ and ‘Boufeggous’ date cultivars [17] were quantified in this investigation at three different times during storage based on two prior drying techniques (infrared and convective) and on the basis of two relative humidity levels (Figure 2).

In both ‘Mejhoul’ and ‘Boufeggous’ cultivars, six phenolic compounds were elucidated as predominant: Chlorogenic-acid derivatives, Ferulic acid, Quercetin-xyloside, Quercetin-derivatives, Quercetin-rhamnoside, and Gallic acid.

The rate and the significance of changes in the phenolic compounds presented in Figure 2 showed non-similar patterns during the cold storage at the two selected relative humidity levels. For the “Quercetin-Rhamnoside”, a general increase was observed (significant or not, depending on the cultivar, prior drying treatment, and relative humidity) in the content of this phenolic compound after four months of storage before regaining approximately the initial concentration at M8. This general trend was recorded for all treatments besides the infrared-dried ‘Mejhoul’ stored at 55% or 65% of RH. The content of “Quercetin-derivatives” showed a significant decrease between the beginning (M0) and the end of storage (M8) for all treatments with the exception of “M-IRD55” and “M-IRD65”. For these two treatments, the amount of “Quercetin-derivatives” remained in the range of 3.01–3.42 g 100 g^−1^ DM without any significant changes (*p* > 0.05). For “Quercetin-Xyloside”, a significant decrease in the eight samples considered by this investigation was observed. Interestingly, Quercetin-Xyloside concentration decreased by about 400% between M0 and M4 for the case of convective dried ‘Mejhoul’ cultivar stored both at 55% and 65% of RH. The rate of decrease for ‘Mejhoul’ dried using infrared drying was about 300% between M0 and M4. Moreover, no significant changes were observed between M4 and M8 for this phenolic compound in the case of all ‘Mejhoul’ treatments. For the ‘Boufeggous’ cultivar, the rate of decrease in Quercetin-Xyloside was less pronounced compared to ‘Mejhoul’. Likewise, the concentration of “Chlorogenic-acid derivatives” (CAD) exhibited a progressive and significant increase during storage for all treatments besides date samples subjected to prior convective drying and stored at 55% of RH: “M-CD55” and “B-CD55”. For these two samples, no significant changes in CAD amount were recorded between M0, M4, and M8. For the “Ferulic acid”, no clear tendency could be pronounced among the majority of treatments. Nonetheless, convective dried ‘Boufeggous’ samples stored at both 55% and 65% of RH showed stable content of Ferulic acid between M0 and M4, before a significant degradation in the concentration of this phenolic acid between M4 and M8. The magnitude of degradation ranged from 4.41 to 3.72 and 3.35 g 100 g^−1^ DM for “B-CD55” and “B-CD65”, respectively. In the case of “Gallic acid”, a gradual and significant increase was observed in all date samples stored under the two RH conditions. The rate of increase was more evident in the case of ‘Boufeggous’ cultivars and especially between the 4th and the 8th month of cold storage. During this period of storage, the rate of increase was around 50%.

### 3.4. Physical Attributes

#### 3.4.1. Weight Loss

**Figure 3:**
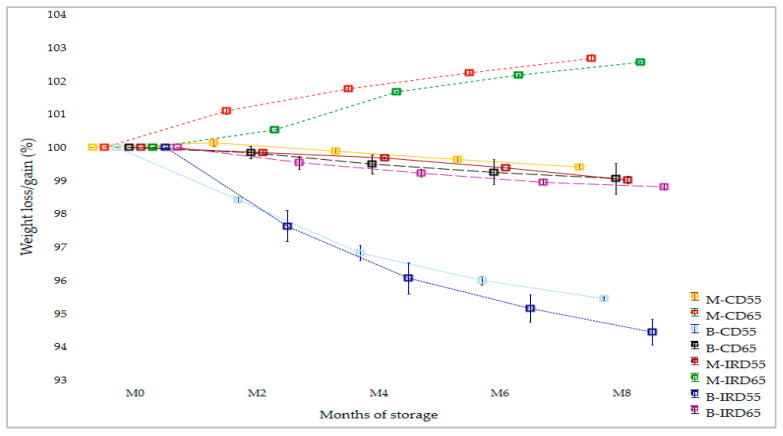
Weight loss changes of ‘Mejhoul’ and ‘Boufeggous’ cultivars subjected to prior convective and infrared drying and stored at two levels of RH during 8 months of storage.

In Figure 3, the curves of weight loss were reported.

As expected, weight loss was related to the initial water activity of date cultivars and to the RH applied during storage. Thus, both convective and infrared ‘Boufeggous’ samples stored at the lower RH of 55% showed a significant decrease during storage, resulting in estimated losses of 5.6% and 4.5% at the end of storage for “B-IRD55” and “B-CD55” treatments, respectively. In contrast, a significant weight gain was observed for ‘Mejhoul’ stored at high RH of 65% reaching a final value of 102.5% and 102.7% for “M-CD65” and “M-IRD65” respectively. Accordingly, the weight losses generated for the four abovementioned treatments exhibited a potential impact of relative humidity applied during storage and also the initial water activity of each date cultivar (Table 1). Also, the rate of change was more pronounced in the first four months of storage (M0 to M4) compared to the last four months (M4 to M8). For the other four treatments (“B-IRD65”, “B-CD65”, “M-IRD55”, and “M-CD55”), a slight change was recorded for weight loss.

#### 3.4.2. Color

**Figure 4:**
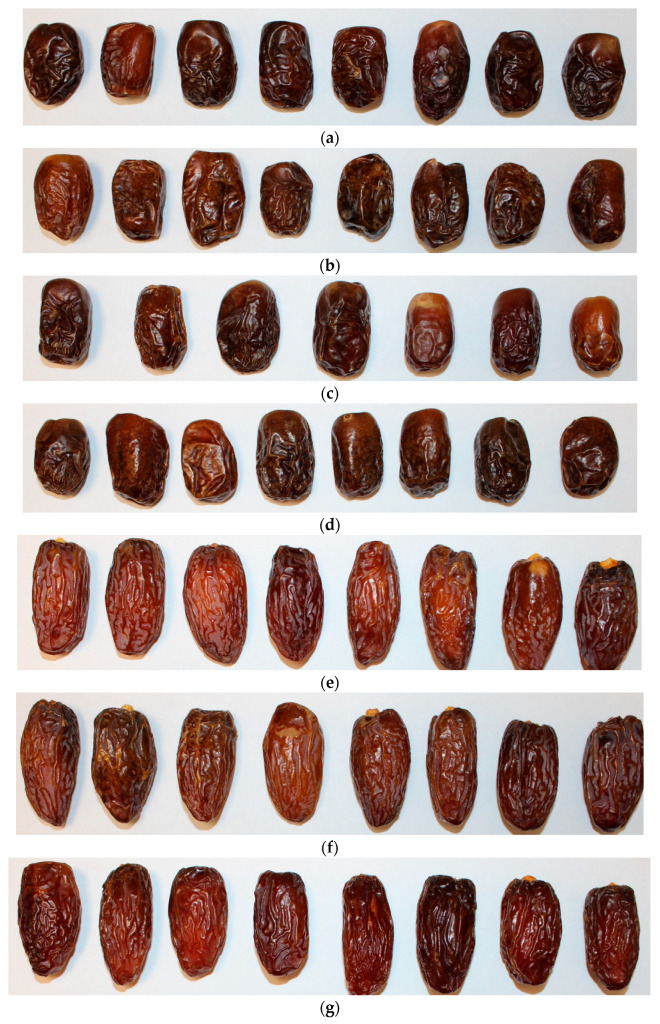

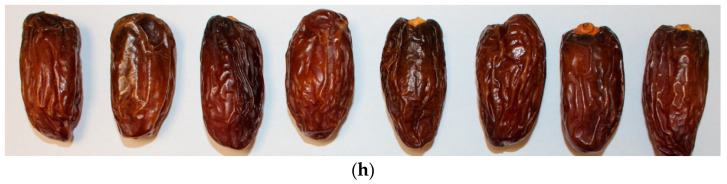
Illustration of stored date fruit. (**a**): B-CD55; (**b**): B-CD65; (**c**): B-IRD55; (**d**): B-IRD65; (**e**): M-CD55; (**f**): M-CD65; (**g**): M-IRD55; (**h**): M-IRD65.

Color is an important sensory attribute that highly impacts consumer preferences since it indicates the level of freshness and total quality. After cold storage, the stored date fruit reflected a similar appearance among all treatments (Figure 4).

**Figure 5:**
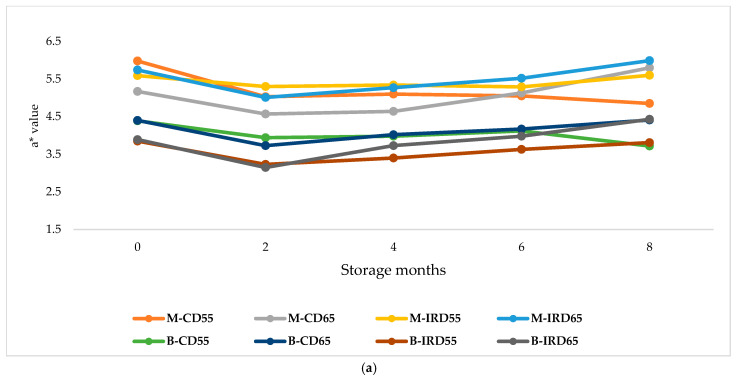

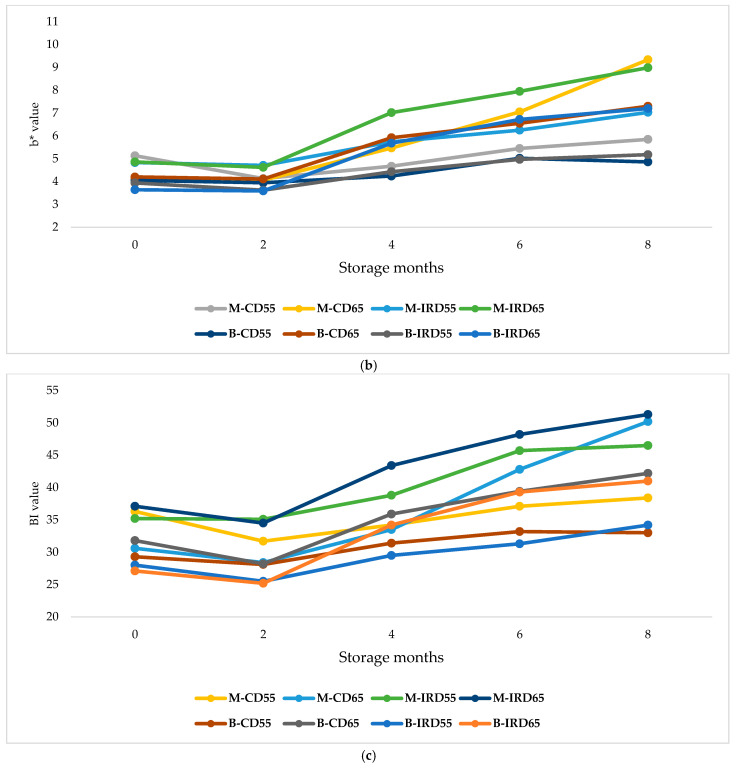
Patterns of color changes during cold storage. (**a**): a* value (red-green); (**b**): b* value (Yellow-Blue); and (**c**): BI value (Browning index).

The variation and the progression of chromatic color changes based on a*, b*, and BI attributes are illustrated in Figure 5. In this investigation, the colorimetric assessment of ‘Mejhoul’ and ‘Boufeggous’ date samples under different storage conditions revealed significant differences in evaluated color attributes. Additionally, a clear pattern was observed during storage among all treatments. For a* (red-green), decreased values were recorded between M0 and M2 for all treatments. Then, a slight and progressive increase until the end of storage to reach approximately the initial value at the beginning of cold storage was observed, with the exception of convective dried ‘Mejhoul’ and ‘Boufeggous’ kept at 55% of RH.

For b*, a stability in the value of this feature was observed between the beginning and the second month of storage. At that point, a slight and progressive increase in b* value was exhibited for all treatments until M8. This pattern was intense for the case of M-CD65 and M-IRD65, which showed a two-fold increase from 4.5 to 9 between M2 and M8. A similar pattern was indicated in the changes of BI as it was described for b*, demonstrating a progressive browning among all stored categories of date fruit. The higher ranges of variation in the browning index were observed for the case of M-CD65 and M-IRD65, which increased respectively from about 32 to 50 for M-CD65 and from 37 to 51 for the case of M-IRD65. Conversely, the smallest changes in BI were recorded for ‘Boufeggous’ cultivar stored at 55% of RH: “B-IRD55” and “B-CD55”. In the case of B-IRD55, the increase between M0 and M8 was significant, while no significant changes were exhibited in BI values during storage for B-CD55.

#### 3.4.3. Hardness

**Figure 6:**
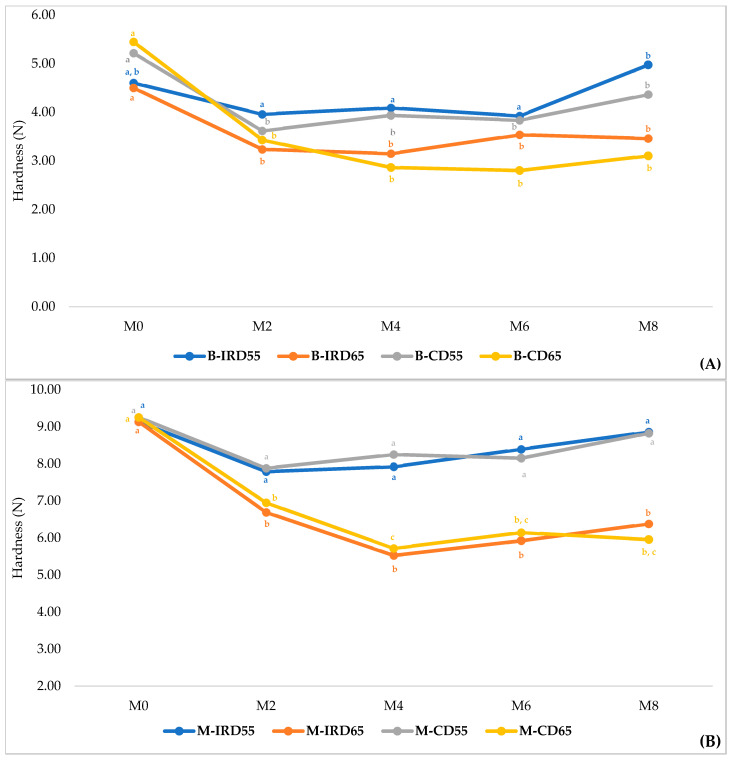
Evolution of Hardness during cold storage for ‘Boufeggous’ (**A**) and ‘Mejhoul’ (**B**) cultivars. For each curve, different letters refer to significant difference (among months of storage) by Tukey test (*p* < 0.05).

As reported in Figure 6, the changes in hardness were partially similar among the eight treatments considered for this study. The general pattern showed a continuous decrease until M2 for both ‘Boufeggous’ and ‘Mejhoul’ cultivars stored at 55% of RH before a slight and continuous increase until M8. For the case of date samples stored at 65%, the decrease in hardness was progressive until M4, when the minimal values were reached before fluctuating between M6 and M8. Under these conditions of storage (65% of RH), the rate of decrease was more pronounced for the ‘Mejhoul’ cultivar: M-CD65 and M-IRD65. Thus, the softening level of ‘Mejhoul’ samples respectively evolved from about 9 N to about 5.5 N between M0 and M4.

Based on cultivar, all ‘Boufeggous’ treatments showed a significant decrease (*p* < 0.05) in hardness between M0 and M2 (with the exception of B-IRD55 treatment). After M2 to the end of storage, no significant changes were recorded for hardness. For the ‘Mejhoul’ cultivar, no significant changes (*p* > 0.05) were exhibited in hardness values for “M-IRD55” and “M-CD55” during the whole period of storage. The hardness ranged between 8 and 9 N for these two treatments. For the other two treatments, a significant decrease from 9 to 6.5 N was noticed since the second month; This numerical decrease continued until the 4th month before starting a slight increase until M8.

### 3.5. Relationship Between Some Quality Attributes and Image Features

**Figure 7:**
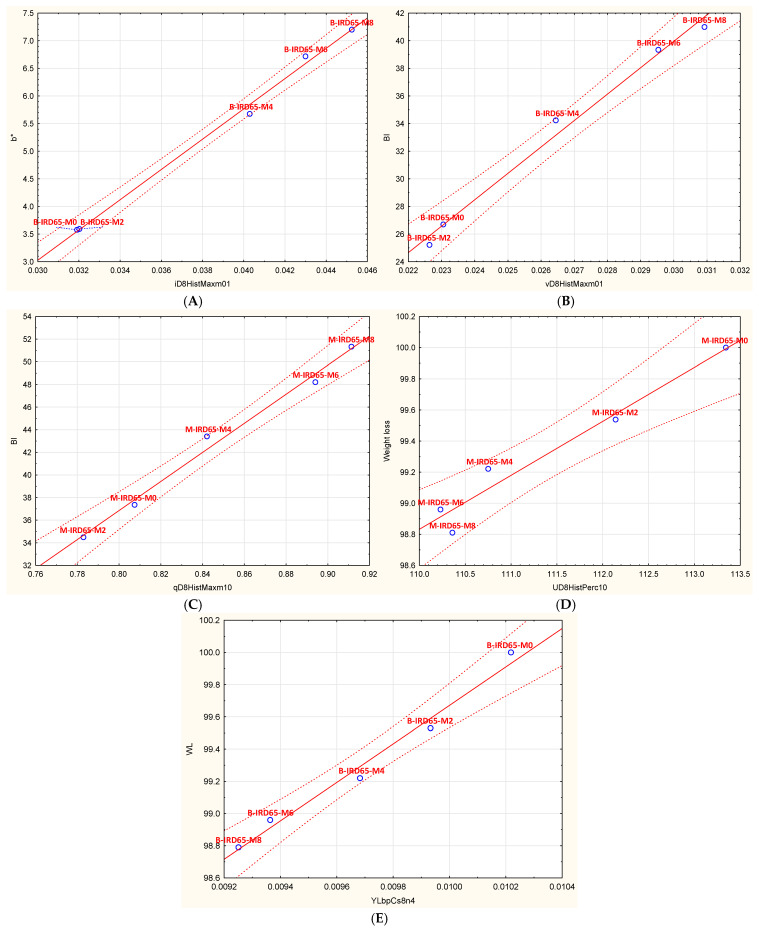
Scatter plots for: b* of B-IRD65 (**A**), BI of B-IRD65 (**B**), BI of M-IRD65 (**C**), weight loss of M-IRD65 (**D**), and weight loss of B-IRD65 (**E**) as correlated with accurate image features.

In Figure 7, weight loss, b*, and BI parameters were correlated with some image features to establish potential correlations.

According to the scatter plot graphs, it is clear that the selected physicochemical attributes correlate with different image textures. Thus, it was found that the b* value for infrared ‘Boufeggous’ kept at 65% of RH correlates with the image feature “iD8HistMaxm01” with a determination coefficient of 0.994. For the browning index (BI), it was indicated that “B-IRD65” correlates with the image feature “vD8HistMaxm01” while the ‘Mejhoul’ cultivar (M-IRD65) correlates with another feature: Qd8HistMaxm10. The coefficient of determination was 0.988 and 0.989 for the two treatments, respectively. Regarding weight loss, which is considered an important indicator for economic losses, this investigation elucidates a high correlation between this parameter and the image feature “UD8HistPerc10” for the case of infrared-dried ‘Mejhoul’ conserved at 65% of RH. For the case of infrared dried ‘Boufeggous’ stored at 65% of RH, the “YLbpCs8n4” was the accurate feature. The determination coefficient was 0.953 and 0.980, respectively. These results indicated that the prediction of weight loss could be possible based only on image features that could be acquired using non-destructive techniques.

## 4. Discussion

The postharvest quality of date fruit (*Phoenix dactylifera* L.) during storage is considerably impacted by several factors such as maturity stage of date fruit, date cultivar, temperature, relative humidity, preharvest treatments such as fumigation, hydration, drying, etc. [11,19]. Such factors determine the behavior of the dates under storage and often lead to insignificant subsequent changes in the main physicochemical attributes of the fruit. In this investigation, drying as an effective and promising pretreatment (prior to storage) was applied using two techniques: convective and infrared drying. Previously, it was suggested that these drying techniques could be applied with the objective of improving the quality of fruit and vegetables during postharvest storage [16]. Accordingly, both convective and infrared drying were combined to specific storage conditions at two levels of relative humidity, which may be considered as an ultimate approach to reduce postharvest weight loss, delay softening, and preserve the functional compounds of date fruit. Also, sugars are among the key intrinsic elements of date fruit. In our study, the changes in sugars based on glucose, fructose, and total sugars were partially similar to previous studies. This dissimilarity could be explained by several complex enzymatic reactions that depend on fruit/vegetable maturity, climate, and storage environment [20,21]. Compared to our findings, it was reported that glucose content was significantly affected by temperature and not by storage time. Nevertheless, fructose was highly affected by storage time, showing an increase in its content as the storage time increased [14]. This pattern was noticed in our investigation only for the ‘Boufeggous’ cultivar stored at lower relative humidity: B-CD55 and B-IRD55. In another investigation, total sugars and reducing sugars were highly affected by temperature and storage period. Thus, it was found that total sugars decreased while reducing sugars were increased or decreased depending on date cultivars [15]. For the ‘Barhi’ cultivar, total sugars showed a significant increase during storage of 80 days at 0 ± 2 °C and RH of 90–95%. The same evolution was exhibited for the content of reducing sugars [22]. The differences between our findings and those revealed previously may be attributed to some extrinsic factors such as temperature, relative humidity, and packaging type; as well as date cultivar, the respiration rate, and the intensity of sugar hydrolysis or biosynthesis [23,24,25].

For phenolic compounds, no comprehensive study was dedicated to assessing the changes in phenolic acids during cold storage of date fruit. All previous works mainly examined the patterns of total polyphenols and flavonoids. Thus, total phenolic content declined gradually for ‘Barhi’ with the progression of storage time. After 60 days of cold storage, some date fruit treated with calcium showed a decrease from about 450 to 260 mg 100 g^−1^ DM [26]. In another study, soft and dried date fruit cultivars kept at +4 °C during 6 months of storage indicated a clear increase in the content of total polyphenols (TP) as well as in total flavonoids (TF) content. In this work, the cluster analysis of TP and TF indicated dissimilar behavior under storage that was explained by the effect of “date type/cultivar” [27]. An in-depth study profiled 11 individual phenolic compounds in ‘Deglet Nour’, with the predominance of “procyanidins” that exhibited (with other major polyphenol groups) a stable content during 9 months of cold storage at 0, 2, and 4 °C [21]. Moreover, ‘Khalas’ and ‘Shishi’ date palm cultivars showed a significant increase in their total phenolic and flavonoid content during storage for 6 and 12 months at +4 °C [28]. Also, chilling at +4 °C for 8 weeks exhibited an elevation by around 218, 180, 60, and 57% in the TP of ‘Safawi’, ‘Mabroom’, ‘Mariami’, and ‘Ajwa’, respectively [29]. The discrepancy among the results found in the polyphenol profile variation with regard to our findings and also between previous investigations could be attributed to date cultivars, complexity of date fruit matrix, polymerization degree of some phenolic acids, and the interactions occurring between sugars and polyphenols [30]. In addition, it is known that the changes in polyphenol profile are related to the activity stimulation of some specific enzymes engaged in the biosynthesis of phenolic compounds [31].

The scarcity and non-availability of results and potential findings explaining the evolution of individual phenolic compounds (as shown in Figure 2) for the case of date fruit (*Phoenix dactylifera* L.) does not allow for a targeted comparison. For other similar stone fruit species, plums showed an increase in chlorogenic acid content during storage, which aligns with our findings reported in Figure 2. The same pattern was indicated for Ferulic acid content of plums, pointing to partially dissimilar behavior compared to our results, which indicate fluctuating changes among storage times [32].

Otherwise, it was illustrated in Figure 2 that ‘Gallic acid’ and “Quercetin-Xyloside (QuX)” were highly influenced by cold storage conditions and indicated opposite patterns in terms of their concentration changes. The decrease found in QuX content among all treatments considered by our study is in agreement with the trend exhibited for two apple cultivars during moderate and long storage [33]. This similar behavior, demonstrating a decrease in QuX content during storage, could be explained by the “superficial scald” phenomenon [33]. This decrease in QuX content could also be explained by its degradation according to a complex mechanism involving some enzymes such as Anthocyanases, peroxidases, and polyphenol oxidases [34,35]. Moreover, one of the potential scenarios explaining the progressive increase in Gallic acid (GA) could be the hydrolytic degradation of tannins. The general biosynthetic pathways leading to the formation of GA are from three precursors: L-Phenylalanine, 3,4,5 trihydroxycinnamic acid, and Shikimic acid [36]. Those hypotheses should be confirmed through an in-depth in silico analysis of the molecular interaction between oxidative enzymes (to be elucidated) of date fruit and polyphenols exhibiting significant changes during storage.

Additionally, firmness is a powerful indicator of fruit quality since soft fruit is highly perishable and more disposed to decay and physical disorders [37]. For the same cultivars ‘Mejhoul’ and ‘Boufeggous’ considered by this study, it was reported that commercial cold storage conditions (+2 to +4 °C) significantly impacted the hardness of both cultivars. Thus, a significant decrease confirming a softening of both cultivars was reported during 3 months of storage from 7.09 to 6.04 N for ‘Mejhoul’ and from 7.92 to 5.95 N for ‘Boufeggous’ [13]. Those findings are in agreement with our results reported in Figure 6 since both cultivars showed a progressive decrease in hardness until M4. However, our findings are in discordance with the patterns elucidated for the ‘Barhi’ cultivar, which indicate a continuous decrease in firmness values during 80 days of storage [22]. In other work, the firmness of ‘Khalas’ date cultivar was significantly affected by cold storage conditions during 6 months without mentioning any clear patterns [38]. Relative humidity was a critical factor that influenced the hardness of cultivars considered by our investigation. Especially, ‘Mejhoul’ kept at higher relative humidity conditions (65%) showed a pronounced decrease in hardness values compared to date samples stored at 55% of RH (panel b of Figure 6). In the literature, it was demonstrated that the mechanical properties of fruit and vegetables are highly associated with relative humidity and temperature conditions during storage [39,40]. In addition, the reduction in hardness may be explained by the breakdown of insoluble solids or by cellular breakdown that led to some membranous porousness [41]. The softening phenomena observed especially for ‘Mejhoul’ samples might be associated with cell wall polysaccharides degradation during storage, as well as with the depolymerization of water-soluble pectin, acid-soluble pectin, and soluble hemi-cellulose. In pineapple, this phenomenon was linked directly to substantial modification in pectin and hemicellulose fractions [42].

For color attributes, our findings given in Figure 5 indicated clearly a significant increase in b* and BI values for both cultivars under all storage conditions, while a* represented a fluctuating trend. Contrary to this, it was mentioned that b* for ‘Barhi’ cultivar followed a progressive decrease during storage. For the same cultivar, a* values increased during the 40 days of storage [43]. The main reason that could justify these differences is that the ‘Bahri’ cultivar is characterized by a yellow skin color compared to the brown color of ‘Boufeggous’ and ‘Mejhoul’. In another investigation, the yellowness attribute (b*) increased in a similar way to the observed trend indicated in Figure 5. Thus, b* increased from 8.32 at the beginning of storage to 10.38, 10.56, and 10.70 at 15, 30, and 45 days of ‘Barhi’ cultivar storage [44]. Overall, the increase in b* (yellow-blue scale) but also the browning index (BI) for the case of date fruit is a powerful indicator of “browning”/”darkening”, which is qualified as a non-suitable and unfavorable phenomenon that could compromise the consumer acceptability [45]. Interestingly, it is confirmed through our study that high relative humidity under storage conditions exhibited higher values of BI for both cultivars (Figure 5). In the literature, external factors were reported to explain this darkening; mainly, storage temperature, relative humidity of storage conditions, time, and the oxygen partial pressure [11]. The browning reactions involving enzymatic and non-enzymatic mechanisms (principally the Maillard reaction), which associate amino acids (and proteins) with simple sugars (as glucose and fructose), often lead to color deterioration during storage. Also, the enzymatic browning is caused by the oxidation of polyphenolic compounds. The two main enzymes responsible for Browning are polyphenol oxidase and peroxidase, which catalyze the oxidation of the -OH functional group into quinones and allow the synthesis of Melanin that contributes to the formation of dusky color [46].

Weight loss (WL) is one of the major causes of fruit deterioration and often leads to important qualitative and quantitative economic losses [47]. In our study, the changes of WL during storage allowed for distinguishing two blocks: the first block included slight changes during storage; the second block consisted of stored date samples that exhibited significant and high weight loss/gain (M-IRD65, M-CD65, B-IRD55, and B-CD55). Accordingly, it could be concluded that date fruit samples with high water activity (case of Boufeggous) showed a significant weight loss when stored at lower RH (55%). Conversely, date fruit with lower a_w_ showed a significant weight gain under high RH conditions (65%). Similarly to our findings, weight loss was found to be gradually and significantly increased during the storage for ‘Barhi’ cultivar [22,48]. Fruit weight loss is considered a complex process implicating the interaction of crop physiology, morphology, and environmental factors, such as the pressure deficit between stored fruit and the surrounding atmosphere. Weight loss is mainly affected by transpiration, and over 97% of WL in fruit and vegetables is due to transpiration that negatively correlated to relative humidity [43,49,50,51]. Also, structural characteristics in outer skin morphology and tissue, as well as the variation in the micro-cracks (count, size, and shape), were found to be important parameters in WL variations. Thus, the exposure of fruit to high relative humidity during storage promotes enlargement and micro-cracking of the fruit surfaces. The micro-cracking is stimulated by genetic traits, orchard management practices, and environmental factors [52]. Accordingly, and besides the cultivar effect, the differences observed in weight loss could be mainly attributed to the physiological factors as detailed previously.

Ultimately, the “cultivar aspect” is an important factor that could explain the storage behavior based on the predefined quality characteristics. As mentioned in Table 1, substantial differences were reported in the total polyphenols and total flavonoids between the two cultivars for the same drying technique. In addition, the water activity was higher in ‘Boufeggous’ compared to ‘Mejhoul’, which resulted in different patterns in terms of weight loss and softening (evaluated in this study through hardness) during storage. In a connected study, the mapping of density (according to a tomographic analysis) between the two studied cultivars revealed that the flesh of the ‘Mejhoul’ cultivar is characterized by high empty spaces compared to ‘Boufeggous’. Also, a different density distribution from skin to seed for each cultivar was exhibited [16].

The predictive potential of image analysis coupled to machine vision (CV) and artificial intelligence (AI) is gaining popularity in the field of food processing and storage. Thus, image features obtained in a non-destructive manner are replacing tedious, destructive, costly, and time-consuming methods used to monitor and evaluate the quality and storability of food matrices. In the specific case of date fruit, image analysis was employed to monitor the external quality of date fruit during frozen storage [18], and also to link some physicochemical and functional compounds with image features [9]. Additionally, many applications of AI and CV were found to be effective in the recognition and maturity assessment of date fruit, as well as in varietal classification. Another important field of image analysis is sorting, grading, and quality inspection of date fruit [53].

## 5. Conclusions

In terms of conclusion, this investigation showed that the application of drying techniques prior to storage impacted the behavior of date fruit under storage. Also, the relative humidity influenced the phenolic compounds and sugar content during storage. Generally, the low RH (55%) could be suggested to preserve the functional compounds and visual quality based on color attributes. However, the weight loss at this low RH could be considered a critical aspect for industrials. The evolution of weight loss/gain was strongly related to the initial water activity of the date fruit cultivar and also to the applied level of RH. The browning index indicated a significant increase during storage, especially for high relative humidity conditions (65%). Interestingly, this investigation indicated that the prediction of date behavior during storage could be elucidated using nondestructive features based on image analysis. From the perspectives, other technologies of preservation, mainly edible coating and controlled/modified atmosphere, could be tested on date fruit to evaluate their effectiveness on maintaining (at least) weight losses and browning index at reasonable levels. Also, it will be relevant to include specific sensory evaluation and explore the biochemical and reaction mechanisms that could explain the significant changes in some phenolic compounds during storage. Moreover, future investigations could consider a wide range of RH control and analyze more date cultivars with high variability in terms of origin, category (soft, dry, etc.), sugar profile (inverted sugars or not), etc.

## Figures and Tables

**Table 1:** Selected physicochemical attributes of date fruit.

	Convective Dried ‘Mejhoul’	Infrared Dried ‘Mejhoul’	Convective Dried ‘Boufeggous’	Infrared Dried ‘Boufeggous’
Total polyphenols (TP) (g 100 g^−1^ DM)	37.1 ± 0.56 ^a^	31.7 ± 1.05 ^b^	29.9 ± 0.77 ^a^	24.9 ± 0.75 ^b^
Total flavonoids (TF) (g 100 g^−1^ DM)	14.9 ± 0.88 ^a^	12.2 ± 0.32 ^b^	7.85 ± 0.86 ^a^	6.19 ± 0.45 ^b^
Total phenolic acids (g 100 g^−1^ DM)	22.2 ± 1.19 ^a^	19.4 ± 0.74 ^b^	22.0 ± 0.70 ^a^	18.7 ± 0.83 ^b^
Glucose (g 100 g^−1^ DM)	41.6 ± 0.52 ^a^	41.9 ± 0.22 ^a^	40.0 ± 0.14 ^a^	39.5 ± 0.38 ^b^
Fructose (g 100 g^−1^ DM)	40.1 ± 0.20 ^a^	40.4 ± 0.55 ^a^	39.3 ± 0.30 ^a^	38.7 ± 0.26 ^a^
Water activity	0.561 ± 0.01 ^a^	0.568 ± 0.01 ^a^	0.634 ± 0.02 ^a^	0.647 ± 0.02 ^a^
Total Soluble Solids (°Bx)	78.4 ± 0.36 ^a^	79.7 ± 0.27 ^b^	73.2 ± 0.19 ^a^	73.4 ± 1.43 ^a^
Hardness (N)	9.26 ± 3.38 ^a^	9.13 ± 4.07 ^a^	5.45 ± 2.32 ^a^	4.50 ± 1.42 ^b^
L*	27.3 ± 2.94 ^a^	26.6 ± 2.99 ^b^	25.7 ± 2.66 ^a^	25.4 ± 2.62 ^a^
a*	5.57 ± 0.17 ^a^	5.66 ± 0.19 ^a^	4.49 ± 0.21 ^a^	3.87 ± 0.18 ^b^
b*	4.54 ± 0.23 ^a^	4.85 ± 0.23 ^a^	4.13 ± 0.21 ^a^	3.79 ± 0.18 ^a^

For each quality attribute and for each cultivar, values with different letters in lines are significantly different by Tukey test (*p* < 0.05).

**Table 2:** Assessment of sugar profile during storage.

	Storage Time (Months)	M-CD55	M-CD65	M-IRD55	M-IRD65	B-CD55	B-CD65	B-IRD55	B-IRD65
Glucose (g 100 g^−1^ DM)	0	41.6 ^a^	41.6 ^a^	41.9 ^a^	41.9 ^a^	40.0 ^a^	40.0 ^a^	39.5 ^a^	40.0 ^a^
	4	40.6 ^b^	40.5 ^b^	40.7 ^b^	39.2 ^b^	40.8 ^b^	39.0 ^b^	41.0 ^b^	39.6 ^a^
	8	42.1 ^a^	40.4 ^b^	42.3 ^a^	40.1 ^b^	42.1 ^c^	41.0 ^c^	42.8 ^c^	41.0 ^a^
Fructose (g 100 g^−1^ DM)	0	40.2 ^a^	40.2 ^a^	40.4 ^a^	40.4 ^a^	39.3 ^a^	39.3 ^a^	38.7 ^a^	39.3 ^a^
	4	38.5 ^b^	37.9 ^b^	38.5 ^b^	38.4 ^b^	38.8 ^a^	39.0 ^a^	40.3 ^b^	39.6 ^a^
	8	39.9 ^a^	38.1 ^b^	40.2 ^a^	38.8 ^b^	41.2 ^b^	39.7 ^a^	41.7 ^c^	39.1 ^a^
Total Sugars (g 100 g^−1^ DM)	0	82.8 ^a^	82.8 ^a^	83.5 ^a^	83.5 ^a^	80.2 ^a^	80.2 ^a^	78.8 ^a^	80.2 ^a^
	4	80.2 ^b^	79.5 ^b^	80.4 ^b^	78.6 ^b^	80.3 ^a^	79.0 ^a^	82.2 ^b^	80.3 ^a^
	8	83.3 ^a^	79.8 ^b^	83.8 ^a^	80.1 ^b^	84.5 ^b^	81.7 ^b^	85.6 ^c^	81.2 ^a^

For each sugar attribute, values with different letters in columns are significantly different by Tukey test (*p* < 0.05).

## Data Availability

The original contributions presented in this study are included in the article. Further inquiries can be directed to the corresponding authors.
